# Machine Learning and Virtual Screening Methods to Discover Potential Cyclin-Dependent Kinase 2 (CDK2) Inhibitors

**DOI:** 10.3390/ph19071019

**Published:** 2026-06-30

**Authors:** Shailima Rampogu, Thananjeyan Balasubramaniyam, Jacek Z. Kubiak, Keun Woo Lee

**Affiliations:** 1 Cachet Big Data Lab, Hyderabad 500045, Telangana, India; shailima.rampogu@gmail.com; 2 Laboratory of Molecular Oncology and Innovative Therapies, Military Institute of Medicine—National Research Institute, Szaserow 128, 04-141 Warszawa, Poland; dhanaj8@gmail.com; 3 Dynamics and Mechanics of Epithelia Group, Institute of Genetics and Development of Rennes (IGDR), National Centre for Scientific Research (CNRS), Faculty of Medicine, University of Rennes, UMR 6290, 35043 Rennes, France; 4 Korea Quantum Computing (KQC), 55 Centumjungang-ro, Haeundae, Busan 48058, Republic of Korea

**Keywords:** CDK2, machine learning (ML), molecular docking, virtual screening, PubChem fingerprints (FPs)

## Abstract

**Background**: Cyclin-dependent kinase 2 (CDK2) is a key regulator of cell cycle progression and an important therapeutic target in cancer treatment. This study aims to identify novel CDK2 inhibitors using an integrated computational approach combining machine learning and structure-based methods. **Methods**: A computational pipeline was developed incorporating Lipinski’s Rule of Five filtering, machine learning (ML)-based activity prediction, molecular docking, and molecular dynamics simulations (MDs). A dataset of CDK2 inhibitors with IC50 values was retrieved from ChEMBL, and molecular fingerprints were generated using PaDEL. A 5-fold stratified cross-validation approach was applied to train multiple classifiers, with the random forest model showing the best performance. Predicted active compounds from the InterBioScreen database were subjected to docking against CDK2 (PDB ID: 2FVD) using PyRx, followed by 100 ns MDS for stability analysis. **Results**: The random forest classifier achieved an AUC-ROC of 0.90 and an accuracy of 0.84. A total of 187 compounds were predicted as active. Among these, two compounds, STOCK4S-00019 and STOCK4S-00025, demonstrated docking scores comparable to the co-crystallized reference ligand. Molecular dynamics simulations confirmed stable binding, consistent interaction patterns, and favorable conformational behavior throughout the simulation period. **Conclusions**: The identified compounds, STOCK4S-00019 (hit1) and STOCK4S-00025 (hit2), show strong potential as CDK2 inhibitors. These findings support their further investigation through experimental validation and highlight the effectiveness of integrated computational approaches in anticancer drug discovery.

## 1. Introduction

Cancer occurs when cells grow uncontrollably and invade other parts of the body. DNA damage causes cancer cells to arise from normal cells. Cancer cells often fail to repair DNA damage properly, unlike normal cells. Only a small proportion of cancers are inherited through germline mutations. Smoking or environmental factors can also damage DNA. Cancer cells metastasize through blood or lymphatic vessels [1].

Cyclins activate cyclin-dependent kinases (CDKs), which are thought to help the cell cycle. Serine/threonine kinases known as CDKs from complexes with cyclins (CDK/cyclin-complexes) to organize cell cycle progression. There are eight CDKs (30–40 kDa) with approximately 40% sequence similarity and nine cyclins (A–I) in mammals. Cyclin subunits are 30–80 kDa [2]. Mutated cell cycle genes cause uncontrolled proliferation and tumor formation. Modifying the activity of these proteins with small molecules may provide an effective strategy to treat cancer [3]. Cyclin-dependent kinase 2 (CDK2) is one of the most important members of the CDK family, involved in various biological processes and playing a critical role in regulating the cell cycle [4]. CDK2 also phosphorylates and interacts with proteins involved in DNA and RNA metabolism, translation, intracellular transport, signal transduction and DNA damage [4]. It phosphorylates numerous transcription factors. CDK2 is essential for DNA synthesis and modulates G1/S and G2/M progression. CDK2 becomes active when complexed with cyclin A or cyclin E. [5]. Some cancers exhibit abnormal CDK2 activation, and many show elevated levels of cyclins A and E, the regulatory subunits, during oncogenic processes [6] CDK2/cyclin A regulates S-phase progression, and CDK2/cyclin E phosphorylates retinoblastoma protein (Rb) for the G1/S transition [7]. Some cancers have abnormal CDK2 activation, and many have elevated cyclins A and E, the regulatory subunits, during oncogenic processes [5]. However, in the absence of cyclin, CDK2 remains inactive [6]. Due to the aforementioned reasons, CDK2 has become an attractive target for cancer therapeutics.

Human CDK2 consists of 298 amino acids and adopts a bilobal monomeric structure. The N-terminal lobe contains residues 1–85 and consists of a predominantly β-sheet structure with a major α-C-helix. In contrast, the α-helix C-terminal lobe is predominantly α-helix, containing helices α1 through α2, α3, α4, α5, α6, α7, α8, and α9 [7]. The hinge region (residues 81–83) allows the two lobes to rotate without disturbing the secondary structure [6]. The C-terminal lobe contains the phosphorylation and activation segments. The catalytic base (Glu51) is in the N-terminal lobe and activates Thr160 phosphorylation in the T-loop. The activation segment (T-loop) lies between the conserved DFG (Asp145-Phe146-Gly147) and APE (Ala170-Pro171-Glu172) motifs. The inhibitory loop (residues 11–18) is called the G-loop or glycine-rich loop due to its conserved glycine residues [8]. Potential inhibitory sites T14 and Y15 are here. Phosphorylation can occur independently of these residues, dramatically reducing kinase activity [8,9]. Typically, the phosphorylation of Y15 and T14 (to a lower degree) is medicated by human Wee1Hu [9,10]. This inhibitory phosphorylation can occur independently of cyclin binding and precede T160 phosphorylation by CAK, because cyclin binding requires prior activated phosphorylation, and CDC25 dephosphorylation of T14 and Y15 is required for full activation to prevent formation of an inactive, excess-phosphorylated complex. The CDK2/cyclin complex is only fully active when T160 is phosphorylated. Additionally, KAP and PP2C (phosphatases) dephosphorylate monomeric CDK2 [9]. The N-terminal α1 helix has a PSTAIRE motif from residues Pro45-Glu51. This PSTAIRE is essential for cyclin binding [9]. This motif helps the CDK2 to interact with the cyclin [7].

Computational drug discovery is one of the most widely used methods for identifying new drug candidates [11,12]. It encompasses two methods, structure-based drug design (SBDD) and ligand-based drug design (LBDD) [13,14]. In SBDD, the interaction between the X-ray or cryo-EM structure of the target and the bound ligand is analyzed to identify new potential inhibitors [15,16,17]. In LBDD, the features of small molecules that have yielded IC_50_ values for a particular target are exploited [18,19,20]. There is evidence that computationally identified compounds exhibit biological activity [21,22,23]. Recently, artificial intelligence (AI) and machine learning (ML) have been applied to the drug discovery process [24,25,26,27]. In the current investigation, the various computational approaches including AI and ML algorithms have been employed to identify potential CDK2 inhibitors.

## 2. Results

### 2.1. Virtual Screening and Lipinski’s Filtering

The compounds retrieved from the InterBioScreen (IBS) database were filtered using Lipinski’s Rule of Five, yielding 333 compounds. Correspondingly, hit1 and hit2 displayed a molecular weight (MW) of 449.51 g/mol and 443.52 g/mol. The number of hydrogen bond donors for hit1 and hit2 is 2, and the number of hydrogen bond acceptors is 5. The LogP values define the lipophilic nature of a compound. The LogP_o/w_ of hit 1 was 3.68, and hit2 was 2.85. Here, hit2 falls within the permissible range of 1–3 [28], indicating that this compound has balanced solubility and permeability, making it ideal for oral administration. On the other hand, hit1 was predicted to be slightly lipophilic in nature; however, it is still acceptable as previously reported [28] and are moderately soluble [29].

Furthermore, we determined that hit1 had 8 rotatable bonds and hit2 had 7 (Table 1).

**Figure 1:**
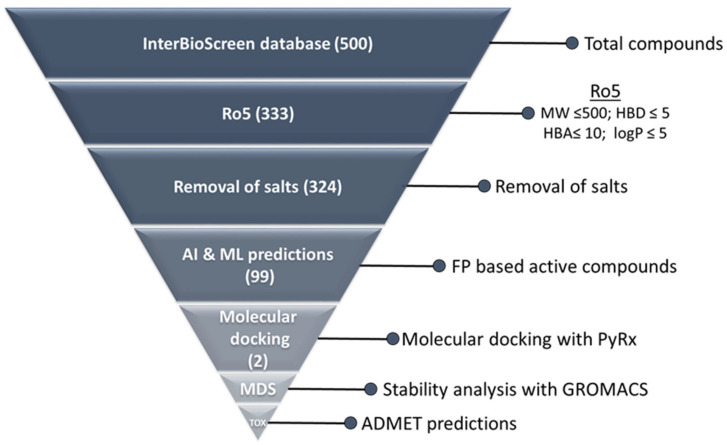
Infographic of virtual screening process to select potential compounds.

We carefully inspected these compounds and removed the salt-containing ones, resulting in 324 compounds. These 324 compounds were subjected to activity prediction based on PaDEL fingerprints (FPs) (Figure 1).

### 2.2. Model Generation and Prediction of New Compounds

**Figure 2:**
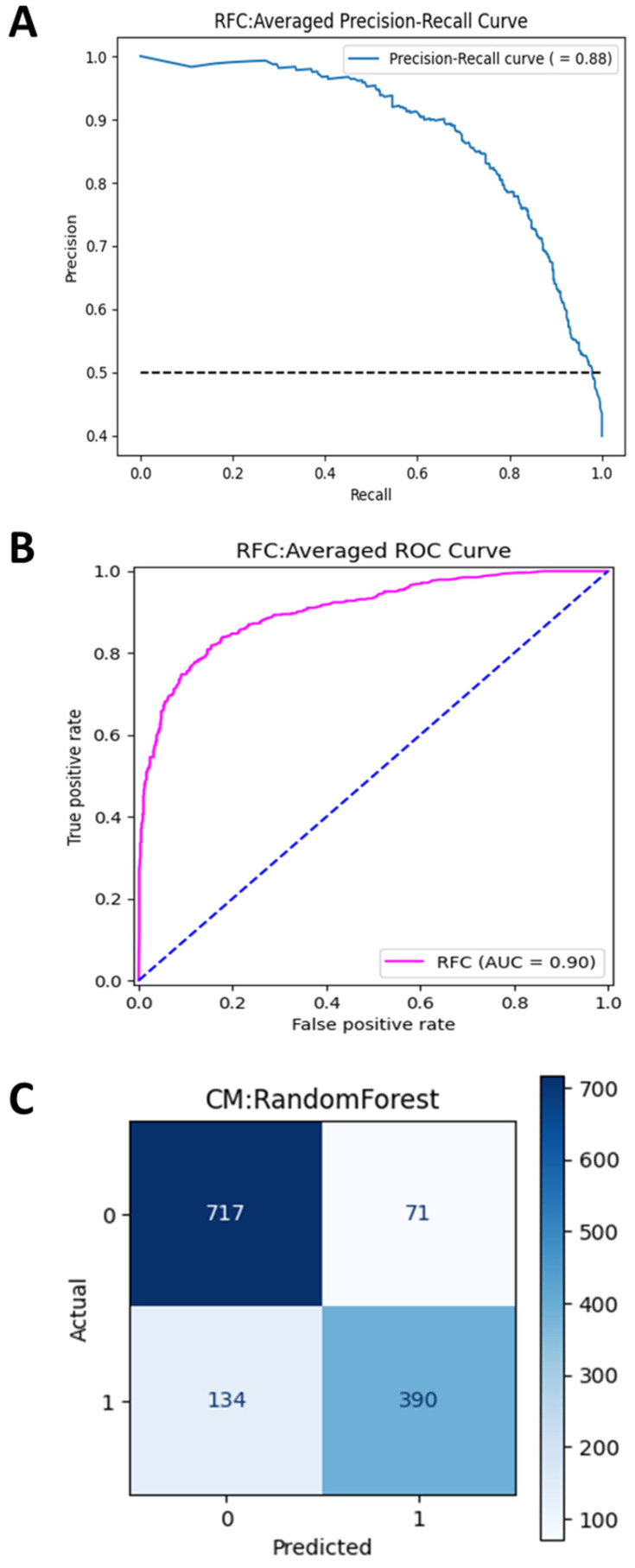
The mean results after 5-fold stratified cross-validation. (**A**) precision-recall curve. (**B**) AUC-ROC curve (**C**) confusion matrix.

Among the various models generated, the random forest classifier (RFC) has the highest performance, with a mean accuracy of 0.84 and a mean AUC-ROC of 0.90 (Figure 2B). Furthermore, the average precision-recall AUC was 0.88 (Figure 2A). The results from the confusion matrix also affirm the superior quality of the ‘rfc’ model. Based on the results, we selected the ‘rfc’ model as the best and used it to assess the prospective activity of the new compounds. We chose this model for further predictions.

The best rfc model was used to predict the activity of the 324 compounds mentioned above. We uploaded the 324 compounds to Google Colab and generated their FPs. The best rfc model was then used to predict their activity Correspondingly, we predicted that 187 compounds would be active and 137 inactive. The active compounds were subjected to molecular docking to elucidate their intermolecular interactions.

### 2.3. Molecular Docking

The molecular docking studies revealed two compounds with binding energies significantly better than those of the reference ligand. The reference ligand exhibited a molecular docking score of −8.1 kcal/mol, while hit1 and hit2 established scores of −9.4 kcal/mol and −9.1 kcal/mol, respectively. Both compounds also interacted with the key residues, thereby adhering to the target’s binding pocket. The binding poses were manually clustered and visualized to highlight key residue interactions. The best poses that met the above criteria were subjected to molecular dynamics simulations (MDSs) to understand the ligand’s behavior within the binding pocket

### 2.4. Molecular Dynamics Simulation (MDS)

The nature of the small molecules in the target’s binding pocket was assessed using GROMACS and analyzed according to various parameters.

#### 2.4.1. Stability Analysis by RMSD

**Figure 3:**
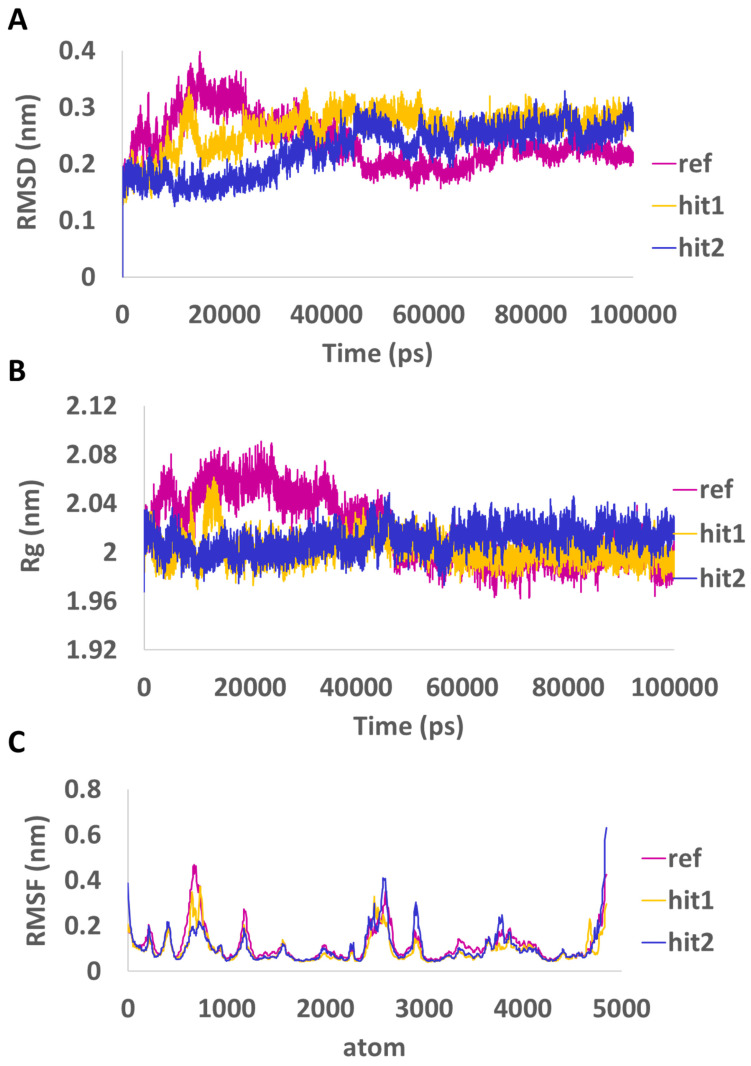
MDS-guided stability analysis. (**A**) RMSD analysis of the three systems. (**B**) Compactness evaluation of the three systems. (**C**) Fluctuation analysis of the three systems.

RMSD measures the deviation of the protein backbone atoms during the simulation trajectory [33]. The results showed that all three systems (reference, hit1, and hit2) were generally stable below 0.3 nm, throughout the simulation, with no significant irregular fluctuations. A minor initial increase was observed within the 20 ns for the reference ligand hit1, after which the systems remained largely stable. In contrast, hit2 demonstrated a highly stable trajectory during the entire simulation. The overall average RMSD for ref, hit1, and hit2 was 0.23 nm, 0.26 nm, and 0.22 nm respectively, indicating that the systems were stable (Figure 3A).

#### 2.4.2. Compactness Evaluation by Rg

The radius of gyration (Rg) profiles indicates the compactness and structural stability of the protein ligand complexes during the simulation [34]. All the systems maintained high compactness with Rg value fluctuating between 2.0 nm and 2.08 nm. Both the reference ligand complex and hit1 exhibited an initial increase in Rg, followed by stabilization after approximately 35 ns and 14 ns, respectively. In contrast, hit2 maintained a highly stable Rg profile throughout the entire simulation trajectory. The mean Rg values for the reference hit1 and hit2 complexes were 2.01 nm, 2.00 nm, and 2.01 nm, respectively, indicating that all systems remained compact and structurally stable.

#### 2.4.3. Fluctuation Analysis by RMSF

The RMSF profiles provide insight into the local flexibility and fluctuations of individual residues during the simulation. Higher RMSF values indicate greater flexibility, whereas lower values reflect higher rigidity and stability. The RMSF profiles of hit1 and hit2 closely resembled that of the reference ligand complex, exhibiting no significant abnormal peaks or fluctuations. This similarity suggests that binding of the hits did not induce destabilizing conformational changes in the protein backbone (Figure 3C).

### 2.5. Number of Hydrogen Bonds

**Figure 4:**
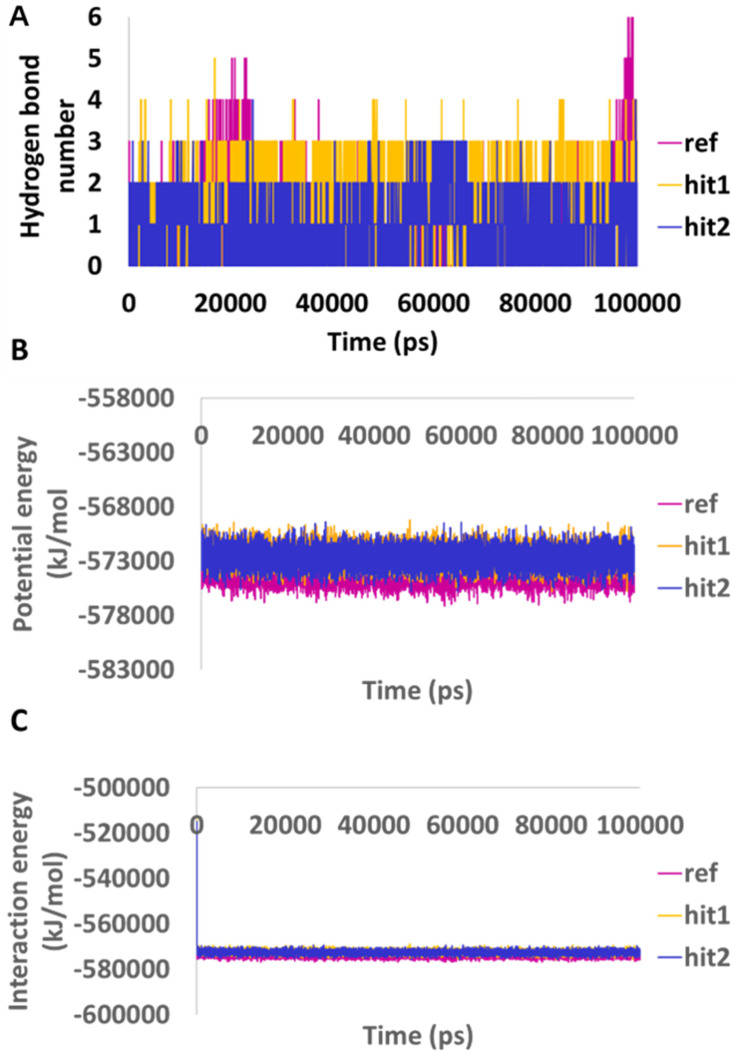
MDS-guided stability analysis. (**A**) Number of hydrogen bonds for the three systems. (**B**) Potential energy analysis. (**C**) Total interaction energy for three systems.

Hydrogen bonding plays a critical role in stabilizing protein-ligand interactions [35]. The average hydrogen bond number for ref was relatively higher at 0.97, while hit1 and hit2 showed hydrogen bond numbers of 1.55 and 1.04, respectively (Figure 4A). This finding indicates that the hits have formed more number hydrogen bonds than the reference.

### 2.6. Potential Energy

The stability analysis was also determined by assessing the potential energy. The potential energy profiles indicate that the systems are perfectly equilibrated and have remained stable throughout the simulation (Figure 4B).

### 2.7. Total Interaction Energy

We calculated the total interaction energy (not the binding free energies) to estimate the strength of the ligand- target interaction. Correspondingly, the ref, hit1, and hit2 have displayed a total interaction energy of −573,894.4662 kJ/mol, 572,363.0412 kJ/mol and −572,534.651 kJ/mol, respectively. This finding shows that the hits display an interaction energy similar to that of the reference, suggesting a relatively similar interaction (Figure 4C).

### 2.8. Binding Mode Analysis

The binding mode reveals how the small molecule has accommodated in the binding pocket of the target and the key interacting residues. Representative structures from the last 20 ns were extracted and superimposed on the X-ray structure, which revealed that the docked compounds occupied a similar binding pocket and displayed a binding mode similar to that of the X-ray structure (Supplementary Figure S1). The compounds were held by multiple key residues, securing them within the binding pocket.

### 2.9. Intermolecular Interactions

Examining intermolecular interactions reveals the key residues that firmly accommodate plausible hits within the binding pocket. Subsequently, we examined the interactions of the ref and the hits

**Figure 5:**
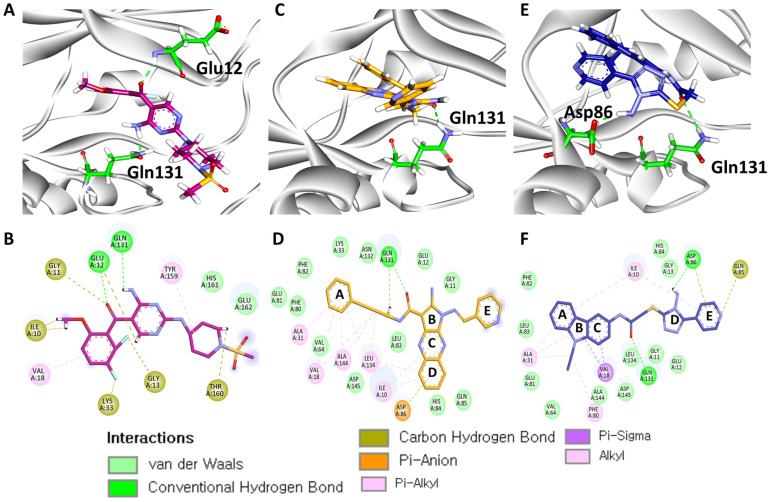
Intermolecular interactions between target and the ligands. (**A**) The hydrogen bond interactions between target and ref. (**B**) Comprehensive interactions between target residues and ref. (**C**) The hydrogen bond interactions between target and hit1. (**D**) Comprehensive interactions between target residues and hit1. (**E**) The hydrogen bond interactions between target and hit2. (**F**) Comprehensive interactions between target residues and hit2.

The ref has formed hydrogen-bond interactions with the key residues, such as Glu12 and Glu131 (Figure 5A), respectively. Furthermore, the carbon–hydrogen bonds were observed with Ile10, Gly11, Glu12, Gly13, Lys33, and The160 residues. The π- alkyl interactions were formed with the residues Val18 and Tyr159 accommodating the ligand in the binding pocket. Additionally, the other key residues including His161 and Glu162 have clamped the ligand in the binding pocket (Figure 5B and Table 2).

Hit1 established a hydrogen bond with the key residue Glu131 (Figure 5C). The same residue has prompted a carbon–hydrogen bond with the ligand holding the ligand at the binding pocket. The residues Ile10, Val18, Ala31, Ala144, and Leu134 have generated π- alkyl and alkyl interactions helping the ligand to be seated in the binding pocket. Ring A has prompted π- alkyl interactions with the residues Val18, Ala31, Leu134, and Ala144. Additionally, Val18 and Leu134 have rendered alkyl interactions with the ligand. Furthermore, ring C and ring D of the ligand have adhered to the target via the π- alkyl interactions. The key residue Asp86 is anchored to the ligand via a π- anion interaction. Besides these interactions, the van der Waals interactions were also observed with the residues Gly11, Glu12, Glu18, Lys33, Val64, Phe80, Leu83, His84, Gln85, Phe82, Asn132, and Asp145, firmly adhering the ligand at the binding pocket of the target (Figure 5D and Table 2).

The hit2 has generated hydrogen bond interactions with the key residues Asp86 and Gly131 (Figure 5E). The residue Gln85 has formed a carbon–hydrogen bond. Furthermore, ring A has generated π- alkyl interactions with Ile10 and Ala31. The Ala31 residue has also interacted with ring B via a π- alkyl interaction. The residue Val18 has prompted a π-sigma interaction with the rings B and C of the ligand. Moreover, Phe80 and Ala31 interact with the ligand via alkyl interactions, holding it in the binding pocket. Additionally, ring E has interacted with Ile10 via a π-alkyl interaction, and the residue Asp86 has generated a π-sigma interaction adhering the ligand at the binding pocket. Additionally, ring E has interacted with Ile10 via π-alkyl interaction, and the residue Asp86 has generated a π-sigma interaction adhering the ligand at the binding pocket. Additionally, the residues Gly11, Glu12, Gly13, Val64, Glu81, Phe82, Leu83, His84, Ala144, and Asp145 form van der Waals interactions, aiding the ligand in being seated in the binding pocket (Figure 5F and Table 2). Overall, these interactions suggest that the hits may act as CDK2 inhibitors. The SMILES of hit1 and hit2 are represented in Supplementary Table S2.

### 2.10. ADMET Analysis

Upon performing the ADMET analysis, it was evident that the compounds have demonstrated acceptable values (Table 1). The compounds have demonstrated a moderate solubility, which is essential for determining their absorption. Furthermore, both hits have been predicted to have high absorption. According to the predictions, the hits cannot cross the BBB and are CYP1A2 inhibitors. The hits are also predicted to be inactive and hepatotoxic, and their bioavailability is within the acceptable range of 0.55 [28].

## 3. Discussion

Cancer is one of the most devastating diseases, characterized by abnormal cell growth originating from various tissues in the body [36]. CDK2 is believed to demonstrate a fundamental role in cancer cell proliferation [37,38]. Broadly, CDK inhibitors act by inhibiting CDK activity, thereby arresting cell cycle progression [39]. The CDK2 harbours two binding sites for inhibitors, including ATP-competitive and non-ATP-competitive sites. The ATP-competitive site exhibits high sequence homology across various CDKs, thereby rendering it less specific or highly toxic. On the other hand, the inhibitors that bind to the non-ATP competitive site aim to exploit interactions between CDK2 and its substrates, thereby achieving greater specificity and efficacy [5]. The ATP-competitive site is located at the interface of N and C domains encompassing the residues from 10 to145. Here, the small molecules compete with the ATP. This site demonstrates hydrophobic features, and a small, highly polar charged heterocyclic compound binds to it. Typically, this site is subdivided into an adenine region, a ribose pocket, a triphosphate-binding region, a hydrophobic region I located opposite the ribose pocket, a hydrophobic region II, and the hinge region. Furthermore, there are two non-competitive binding sites, site II and site III. Site II has amino acids from 97–101, 104, 194, 196–204, 214, 217–218, 246, 250–251, and 253–254. Site III includes amino acids from 124, 152, 154–156, 172, 176–182, 184, 227–230, 232–234, and 270–272. Site III also binds to short peptides that disconnect the CDK2/cyclin E complex [6]. Additionally, the inhibitors that bind to the CDKs at a given conformation may be grouped into four types. Type I (DFG-in) inhibitors bind ATP and compete for the active site. Type II inhibitors accommodate the ATP-binding pocket and stabilise the conformation (DFG-out) that is catalytically inactive. The type III inhibitors occupy the site adjacent to the ATP binding pocket. Type IV inhibitors bind to a pocket far from the ATP binding pocket [40].

ML approaches have obtained inhibitors from large databases [41,42,43]. Different methods employed for drug discovery include AI—powered identification of the target and its validation, virtual screening employing AI, AI methods to predict drug properties and so on [44]. The AL and ML approaches are efficient techniques in the kinase drug discovery that can that can be used to predict inhibitor selectivity, improve lead compounds, and recommend novel candidates with enhanced specificity [45]. In another study, the authors employed feature selection approach for kinase inhibitor classification with ML [46]. Furthermore, a workflow-based approach to identify kinase inhibitor resistance is another benefit of AI [47]. These advanced methods have also been employed in identifying CDK2 inhibitors [48,49,50]. AI-based computational approaches have retrieved dual EGFR-CDK2 kinase inhibitors [51]. Another study has explored in silico and quantum chemistry approaches to discover CDK2 potential inhibitors [52]. A research group has initiated a binding affinity model-based approach to predict and identify compounds that would cause chromosome damage [53]. A study involved cheminformatics technologies together with ML to identify phytocompounds has CDK2 inhibitors [54]. Additionally, there are several research reports that focus on ML and computational methods to discover potential CDK2 inhibitors [55,56,57,58,59,60,61].

The current study identifies two potential inhibitors by applying computational methods. These compounds have demonstrated acceptable ADMET properties and comply with Lipinski’s rule of 5.

Furthermore, FP-based identification of potential inhibitors has been popular in recent times [62,63,64]. The FPs of known active compounds was used to predict FP-based active or inactive compounds, and ML and DL models were built. Accordingly, the selected model was used to predict the bioactivity of the newly discovered compounds. In the current investigation, FPs were used to identify potential active compounds and has shown lower molecular docking scores than the reference. The molecular docking results indicate that the potential inhibitors occupy the same binding pocket as the co-crystallized compound, thus representing the same binding mode as the bound ligand. The intermolecular interactions have demonstrated the establishment of a key residue interaction. Based on intermolecular interactions, it was observed that the hits interact with Ile10, Val18, Ala31, Gly11, Glu12, Glu18, Lys33, Val64, Phe80, Glu81, Phe82, Leu83, His84, Gln85, Asp86, Gln131, Asn132, Leu134, Ala144, and Asp145. These interactions were also observed in the previously [65,66], suggesting that the identified hits might act as CDK2 inhibitors. The hits have formed hydrogen-bond interactions with the key residues, as observed in the X-ray target structure (Supplementary Figure S2).

The hits have formed hydrogen bonds with key residues Gln131 and Asp86, as observed in the X-ray structure, with a carbon–hydrogen bond and a hydrogen bond [67]. These interactions were previously reported [68]. Furthermore, the residue Ile10 forms a π interaction with the hits, as observed in the X-ray structure and in the reported studies [69]. Additionally, the MDS findings have ensured that the compounds have yielded stable RMSD, RMSF, and Rg values.

The investigation further involves comparing computational molecular docking studies of the retrieved compounds with those of the three known inhibitors, CVT-313, roscovitine, and dinaciclib [70,71,72]. The molecular docking studies showed that the three compounds yielded higher docking scores than the retrieved compounds. Correspondingly, the compound CVT-313 yielded a molecular docking score of −7.5 kcal/mol, the compound roscovitine yielded a molecular docking score of −7.8 kcal/mol, and dinaciclib yielded a molecular docking score of −8.6 kcal/mol, respectively. Furthermore, these compounds have established hydrogen-bond interactions with key residues, including Asp86, Gly131, and Asn132. Additionally, several other residue interactions were observed, as seen in the hits. These results illustrate that the identified hits may be potential CDK2 inhibitors (Supplementary Table S1).

The novelty of the retrieved compounds was ensured by evaluating them in PubChem using SMILES as input. It was observed that these compounds were not analysed against the CDK2 target, thereby demonstrating their novelty, and could be further evaluated in vitro. The 2D structures of the hits are given in Table 1.

Although the present study is entirely computational, experimental validation is a critical next step to confirm the predicted inhibitory activity of STOCK4S-00019 and STOCK4S-00025 against Cyclin-dependent kinase 2 (CDK2) [73,74]. The integrated workflow employed herein, including machine-learning classification (AUC = 0.90), molecular docking, molecular dynamics simulations, and ADMET profiling, was designed to minimize false-positive predictions and to prioritize high-confidence candidates for subsequent biological evaluation. Nevertheless, enzymatic and cellular assays remain essential to verify direct target engagement and functional inhibition. To this end, in vitro CDK2 kinase inhibition assays using the recombinant CDK2/cyclin E complex are recommended to directly measure enzymatic activity. Determination of IC_50_ values through ATP-competitive enzymatic assays would confirm potency and binding at the ATP pocket predicted by docking analysis [75].

In addition to biochemical validation, cell-based studies are necessary to assess functional relevance in a biological context. Cell viability and proliferation assays in CDK2-dependent cancer cell lines would determine antiproliferative effects, while flow cytometry–based cell-cycle analysis could confirm G1/S phase arrest consistent with CDK2 inhibition [76]. Furthermore, Western blot analysis of phosphorylated retinoblastoma (Rb) protein would validate suppression of downstream CDK2 signaling pathways. These experimental approaches provide a clear translational roadmap for validating the computational predictions presented in this work. Therefore, the identified compounds should be regarded as high-priority candidates for in vitro investigation, and the present study serves as a hypothesis-generating framework to guide targeted experimental validation rather than replace biological confirmation.

## 4. Materials and Methods

### 4.1. Selection of the Small Molecules

The small molecules for the present study were retrieved from the InterBioScreen (IBS) database (https://www.ibscreen.com/) accessed on 20 January 2026 [77]. A total of 500 small molecules were considered, which were further filtered based on Lipinski’s rule of 5. Accordingly, the compounds that demonstrate a molecular weight ≤ 500, hydrogen bond donors ≤ 5, hydrogen bond acceptors ≤ 10, and logP ≤ 5 [78]. This rule predicts the oral bioavailability of a given compound [79]. The resultant compounds were upgraded to molecular docking studies to delineate the binding mode and key residue interactions [80,81].

### 4.2. Selection of the Target

The target of the current investigation is the X-ray structure of cyclin-dependent kinase 2 (CDK2) co-crystallized with the inhibitor diaminopyrimidine. PDB ID: 2FVD (hereinafter referred to as ref), with a resolution of 1.85 Å. The protein structure was prepared by dislodging the water molecules. The missing residues were filled in the Discovery Studio Visualiser (version 2025) (hereinafter referred to as DS) by enabling Tools → Macromolecules → Build and Edit Protein. The structure was then refined using the GalaxyWEB server [82]. For molecular docking, the binding site was selected as all atoms within 9 Å of the co-crystallized ligand, with X, Y, and Z coordinates of 1.156700 Å, 28.449967 Å, and 8.538567 Å, respectively. Accordingly, the key residues are marked for residues Ile10, Val18, Ala31, Lys33, Val64, Phe80, Glu81, Phe82, Leu83, His84, Gln85, Asp86, Lys89, Gln131, Asn132, Leu134, Ala144, and Asp145, respectively. The target and ligands were prepared, and molecular docking studies were performed in PyRx after activity prediction using ML and deep learning approaches.

### 4.3. ML and Model Generation

#### 4.3.1. Data Curation

To generate the ML models to eventually predict the active and inactive compounds of the new compounds. For this, compounds that have been experimentally shown to inhibit CDK2 were considered. They were subsequently downloaded from the ChEMBL database. This was done on Google Colab with pandas with the target search ‘CDK2’. The compounds with biological activity reported in ‘nM’ were selected for further evaluation. This resulted in 1862 compounds. Furthermore, the ‘NA’ and the duplicates were removed, resulting in 1312 compounds. These compounds were further divided into active and inactive based on the IC50 values. Accordingly, standard values ≤ 500 nM were labelled as active compounds, and the remaining compounds were labelled as inactive. The cutoff for defining active and inactive compounds is based on previous reports, where compounds with 500 nM are grouped as active [83,84,85]. Another study has classified IC50: <10 μM as active [86]. Generally, 500 nM is equivalent to 0.5 µM, which is considered active [87]. This yielded 788 active and 524 inactive compounds. Subsequently, using the ‘Label Encoder’, a preprocessing method, the active compounds are encoded as ‘0’ and inactive compounds are encoded as ‘1’.

#### 4.3.2. Generation of the Fingerprints (FPs)

The compounds obtained from the above step were subjected to FP generation. From the data, the smiles were retrieved and saved for further analyses to generate the fingerprints (FPs) employing PaDEL FP [88]. Correspondingly, PubChem, Electrotopological State (Estate), AtomPairs2D, Molecular ACCess Systems (MACCSs), and Substructure FP were generated and saved in the CSV format. Correspondingly, of the PaDEL fingerprints generated, MACCSs [89,90] has 166 bits, Estate [91,92] has 79 bits, AtomPairs2D [92,93] has 780 bits, PubChem [91,92,94] has 881 bits, and Substructure has 307 bits. The FPs of all the models were joined prior to the model generation.

#### 4.3.3. Model Generation

After the data were normalized with *StandardScaler* [95], the classification models employed were random forest (RF), eXtreme Gradient Boosting (XGBoost, XGB), KNeighborsClassifier (KNC), logistic regression (LR), decision tree (DT). The model with the high accuracy was saved using ‘pickle’ to predict the activity of new compounds. Each of the models was built using various parameters. The RF algorithm is exclusively proposed for datasets with varied features. It executes by eliminating the outliers, thereby simplifying the data [96]. The main purpose of RF in drug discovery is regression, classification and feature selection [96]. KNC is a supervised lazy learner classification algorithm that executes classification depending on the data neighbors and is uncomplicated to understand [97]. LR is a widely adapted ML algorithm that determines the correlation between a single predictor or numerous predictors. This is broadly of two types, simple regression and multiple regression [98]. For the current study, the LR random state used was 100 and in KNC the n_neighbors were 5, the metric used was ‘minkowski’, and p was selected as 2. The DT is a non-parametric algorithm, the data is divided into sections that look like branches, generating an inverted tree with internal, leaf, and root nodes. This approach can deal with large complex data devoid of applying a difficult parametric framework. The DT can be employed for classification and regression problems [99,100]. In DT the criterion used was ‘entropy’, and random state was selected as 0. In RF, several decision trees are combined, and the predictions are obtained by averaging. This algorithm was initially put forth by L. Breiman and has gained popularity for regression and classification problems. The RF outshines when the count of the variables is more than observations [101]. The parameters for the RF are that the n_estimators is 200, the criterion is ‘entropy’, and the random state is 0. In this method each tree is generated employing several cores, and the data is arranged to reduce processing time [102]. This algorithm employs gradient boosted decision trees for its implementation [103] and is quite popular amongst the researchers due to its exceptional performance [104]. In the current investigation, the parameters for XGB, the n_estimators, are equal to 20. Typically, a 5-fold stratified cross-validation was used [105,106,107,108]. For the present investigation, external validation has not been used. The mean of each parameter is reported.

### 4.4. Evaluation Matrix

Different parameters of the evaluation matrix were employed to assess the performance of the model, such as confusion matrix, accuracy, precision, recall, F1 score, and AUC-ROC.

### 4.5. Confusion Matrix

This renders the ability of the model’s performance, comparing the actual inputs with the predicted inputs. The results are generally displayed in a tabular form with true positives [TP] and true negatives [TN] (correct prediction) and false positives [FP] and false negatives [FN] (inaccurate predictions) [109].

### 4.6. Accuracy

This is defined as the capability of a given model to accurately predict the given instances to the ratio of total instances [109].

Accuracy=TP+TN/TP+TN+FP+FN

### 4.7. Precision

Precision renders information about the efficiency of the model in detecting the actual positives and predicting them as positives [109].

Precision=TP/TP+FP

### 4.8. Recall

Recall, also referred to as sensitivity, is a useful indicator for assessing a model’s ability in predicting positive outcomes [109].

Recall=TP/TP+FN

### 4.9. F1 Score

F1 score is another matrix employed for classification models using recall score and precision score typically their harmonic mean [109].

$$F1\;score=2\times \frac{Precision\times Recall}{Precision+Recall}$$

### 4.10. AUC-ROC

This is one of the finest methods available to develop a robust learning model and can also be utilised to evaluate the learning algorithms. The AUC-ROC helps in ranking the models, and it is regarded as a superior matrix than the accuracy [110].

ROC is a curve with probabilities, while the AUC separates the data according to the labels. Correspondingly, the greater AUC signifies a good model performance [111].

### 4.11. Molecular Docking Studies

The molecular docking studies were conducted employing PyRx that permits the screening of small molecules. The Python programming language is employed to build PyRx [112] and works utilizing Auto Dock Vina, AutoDock and Open Babel [113]. Before initiating the molecular docking, the ref was redocked into the binding pocket. The result showed that the redocked pose was bound in a similar manner as that of the ref, with an RMSD of 1.95 Å (Supplementary Figure S3). Typically, this reading is within the acceptable limits [114,115], implying that the parameters chosen for the molecular docking were reproducible. Subsequently, the prepared target and the ligands were upgraded to molecular docking studies.

### 4.12. Molecular Dynamics Simulations (MDSs)

The molecular dynamics simulation studies were conducted to gain in-depth understanding of how the ligand behaves at the binding pocket of the target. The MDS was conducted employing GROMACS 2018.4 using the CHARMM27 all-atom force field [116]. The ligand topologies were extracted using SwissParam [117]. Correspondingly, the dodecahedron box was generated and solvated with the TIP3P water model, and the *Cl* ions were added. The system was then energy minimized with the steepest descent minimization algorithm. The protein and the ligand were coupled, and a dual equilibration was conducted. The first phase of equilibration was with *NVT* (constant Number of particles, Volume, and Temperature) conducted for 100 ps at 300 K using V-scale thermostat. The second phase of equilibration was executed with NPT (constant Number of particles, Pressure, and Temperature) for 100 ps using Berendsen for 1 bar. This ensemble was upgraded to run a 100 ns MD run under periodic boundary conditions. The results of the MDSs were evaluated using the visual molecular dynamics (VMD) [118] and DS [119,120]. Subsequently, the stability analysis was examined for the protein backbone with root mean square deviation (RMSD), root mean square fluctuation (RMSF), and radius of gyration (Rg) along with the binding mode analysis, protein ligand interaction and interaction energy. It should be noted that, initially, a 20 ns simulation was performed, and after the results stabilized, it was increased to 100 ns (Supplementary Materials).

### 4.13. ADMET Assessment

The final compounds were further assessed for absorption, distribution, metabolism, excretion, and toxicity. ADMET analysis can help to prioritize a given compound [121]. These predictions are conducted employing the SwissADME and ProTox webservers [30,31,32,122]. The parameters calculated are solubility based on ESOL, blood-brain barrier (BBB) penetration, calculating if a compound is a CYP1A2 inhibitor, gastrointestinal (GI) absorption, LogP_o/w_, and hepatotoxicity. While all the predictions are executed utilizing SwissADME, hepatotoxicity was assessed with ProTox. Furthermore, the bioavailability score was also computed, which gives an idea of the complete availability of a drug to the desired target or location [123].

However, it has to be understood that the computational analysis are largely predictions. While the computational analysis establishes the identified hits as potential CDK2 inhibitors, it is essential to validate them in in vitro.

## 5. Conclusions

In this study, an integrated computational approach combining ML, virtual screening, molecular docking, and 100-ns molecular dynamics simulations successfully identified two novel CDK2 inhibitors, STOCK4S-00019 and STOCK4S-00025. Both compounds demonstrated comparable molecular docking to known inhibitors, formed stable interactions with key hinge residues, and maintained favorable ADMET profiles, including high gastrointestinal absorption, moderate solubility, and low toxicity. These findings highlight their potential as selective CDK2 inhibitors.

The identified compounds represent promising scaffolds for further development in oncology. While computational analyses support their efficacy, experimental validation through in vitro and in vivo studies is necessary to confirm their biochemical and cellular activity. The code is publicly available to ensure reproducibility and facilitate further research in CDK2-targeted drug discovery.

## Figures and Tables

**Table 1:** Filtering and ADMET analysis of the discovered hits and their 2D structures.

Parameters	Hit1	Hit2	Ref.
Molecular weight	449.51 g/mol	443.52 g/mol	[30]
No. of hydrogen bond donors	2	2	
No. of hydrogen bond acceptors	5	5	
Rotatable bonds	8	7	
Consensus LogP (1–3 recommended)	3.6	2.85	
Solubility (ESOL)	moderately soluble	moderately soluble	
BBB permeant	no	No	
CYP1A2 inhibitor	yes	Yes	
GI absorption	high	High	
Consensus LogP_o/w_	3.68	2.85	
Hepatotoxicity	inactive	Inactive	[31,32]
Bioavailability Score	0.55	0.55	[30]
2D structures	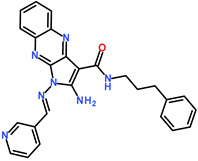	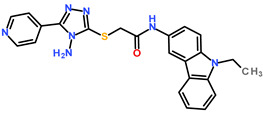	-

**Table 2:** Comprehensive key interactions between the target residues and the ligands.

Compound	Hydrogen Bond	Carbon Hydrogen Bond	Alkyl, π- Alkyl Interactions	van der Waals Interactions
ref	Glu12:HN-lig:O3 Gln131:OE1-lig:H2	Ile10, Gly11, Glu12, Gly13, Lys33, The160	Val18, Tyr159	His161, Glu162
hit1	Gln131:HE21-lig:O1	Gln131	Ile10, Val18, Ala31, Ala144, Leu134	Gly11, Glu12, Glu18, Lys33, Val64, Phe80, Leu83, His84, Gln85, Phe82, Asn132, Asp145
hit2	Asp86:OD2-lig:H2 Gln131:HE21-lig:O1	Gln85	Ile10, Val18, Ala31, Phe80	Gly11, Glu12, Gly13, Val64, Glu81, Phe82, Leu83, His84, Ala144, Asp145

## Data Availability

The original contributions presented in this study are included in the article/Supplementary Materials. Further inquiries can be directed to the corresponding authors. GitHub link: https://github.com/SRampogu/CDK2_ML, accessed on 20 January 2026.

## Supplementary Materials

The following supporting information can be downloaded at: https://www.mdpi.com/article/10.3390/ph19071019/s1, Figure S1: Accommodation of the ligands at the binding pocket of the target; Figure S2: The 2D interactions between the X-ray structure of target and ligand; Figure S3: The redocked result of the ref; Table S1: Molecular docking results of the known CDK2 inhibitors; Table S2: Compounds.
