# Supplementary material for: Machine Learning and Virtual Screening Methods to Discover Potential Cyclin-Dependent Kinase 2 (CDK2) Inhibitors
Source: Pharmaceuticals (Basel). 2026 Jun 30;19(7):1019. doi: 10.3390/ph19071019 (PMC13414861; doi:10.3390/ph19071019)
Supplement: Supplementary file 1 [file pharmaceuticals-19-01019-s001.zip › pharmaceuticals-4145890-supplementary.pdf]

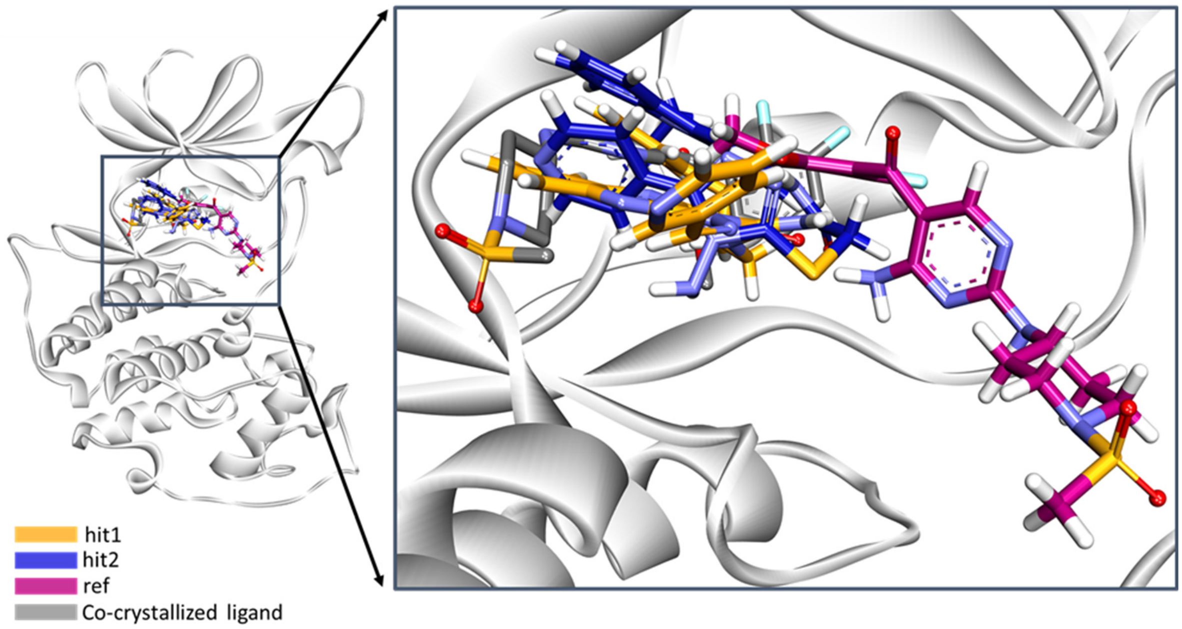

Figure S1: Accommodation of the ligands at the binding pocket of the target. 1A) The ligands are seated at the binding pocket of the pocket. 1B) The zoomed version

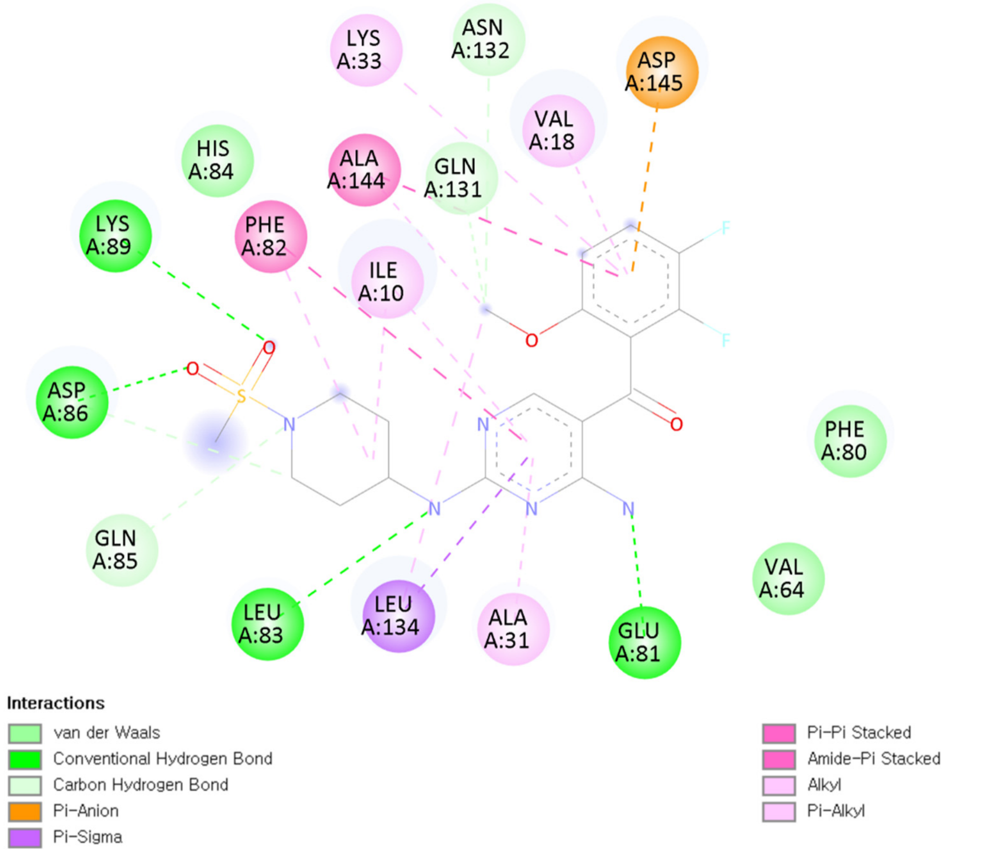

Figure S2. The 2D interactions between the X-ray structure of target and ligand.

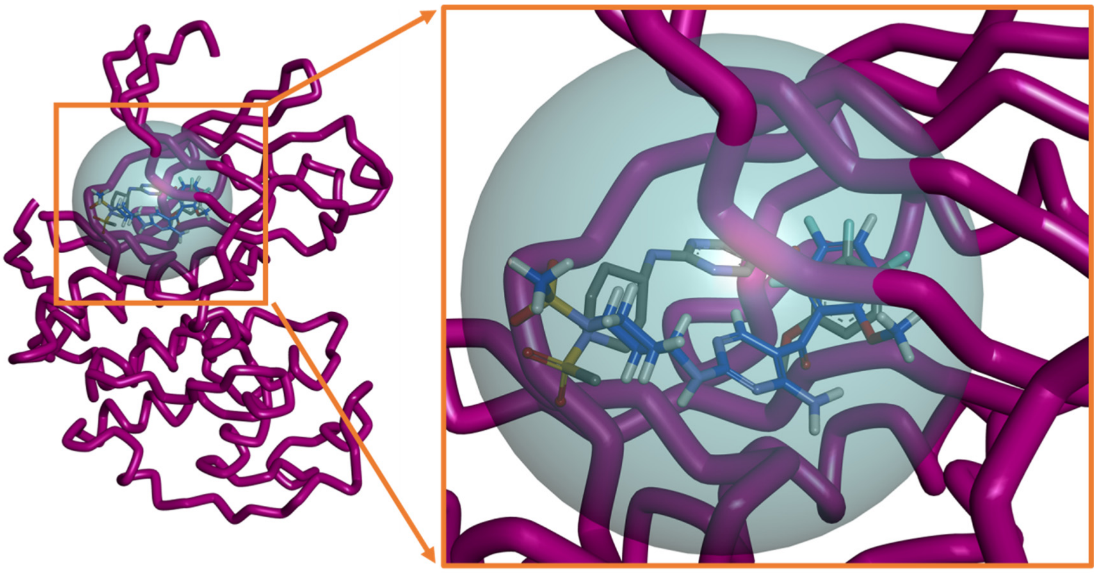

Figure S3:The redocked result of the ref. The redocked pose (blue) occupies the same binding pocket as that of the inbound ligand (grey).

Table S1: Molecular docking results of the known CDK2 inhibitors.

Compound name	Binding affinity score PyRx (kcal/mol)	2D interaction results
CVT-313	-7.5	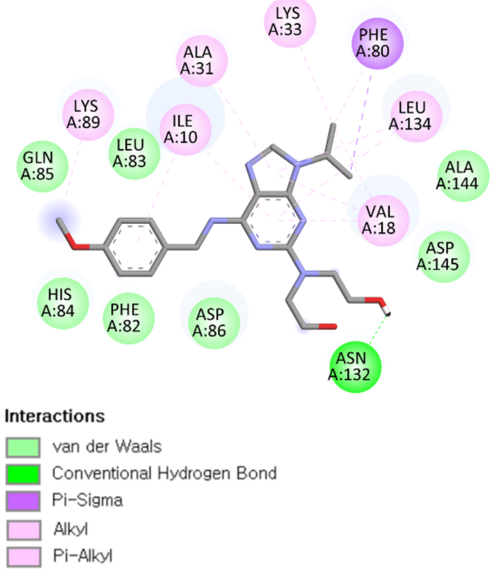

<table>
<tr><td>roscovitine</td><td>-7.8</td><td>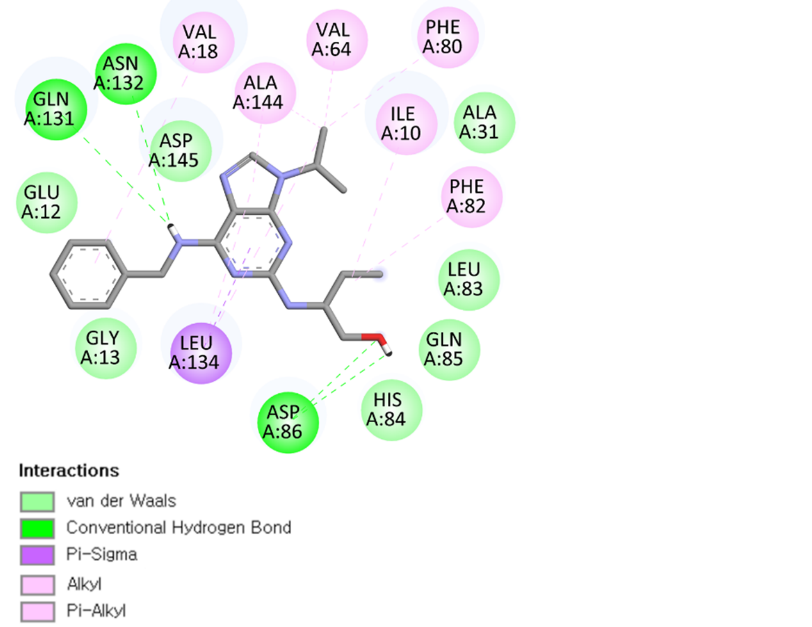
</td></tr>
<tr><td>dinaciclib</td><td>-8.6</td><td>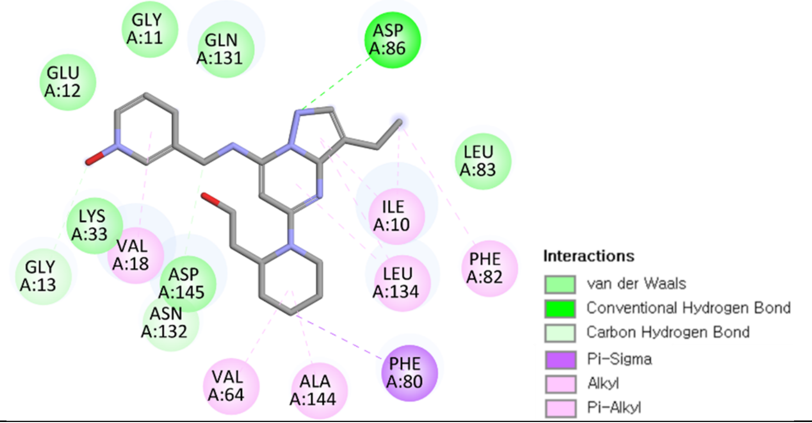
</td></tr>
</table>

Table S2 Compounds.

Compound name	SMILES
STOCK4S-00019	NC=4N(/N=C/C=1C=NC=CC=1)C3=NC=2C=CC=CC=2N=C3C=4C(=O)NCCC/C5=C/C=CC=C5
STOCK4S-00025	NN1C(=N/N=C1/[S]CC(=O)NC=2C=C4C(=CC=2)N(CC)C=3C=CC=CC=34)C5=CC=NC=C5
